# Commentary on a smoke-free medical campus in Jerusalem: data for action

**DOI:** 10.1186/s13584-016-0087-2

**Published:** 2016-06-28

**Authors:** Steven A. Schroeder

**Affiliations:** Distinguished Professor of Health and Healthcare, Department of Medicine, University of California, San Francisco, California USA

## Abstract

Over the past 30 years, Israel has made great progress in attitudes and practices about smoking; probably nothing else has contributed more to the health of its population. Yet, a recent survey about a non-smoking ban at an Israeli health sciences campus found incomplete enforcement. In addition, smoking rates among health sciences students, though lower than the general population, were higher than might be expected based on rates in other developed countries. Whether the ban is—as the authors speculate—“an intrusive life style intervention” or a justifiable public health intervention, cuts to the heart of the history of tobacco control efforts and their opposition by the tobacco industry. Despite concerns that the Israeli population is not ready to accept smoke-free bans, experience in other countries suggests otherwise.

## Background

In 1987 I had the privilege of serving as visiting professor at the Ben Gurion Medical School (now Goldman School) in Beersheva. During that month I frequently accompanied senior internal medicine professors on ward rounds, and also engaged in conversations on diverse medical topics. Although there were many similarities between how medicine was practiced and taught at the Soroka/Ben Gurion hospital and my own institution—the University of California San Francisco (UCSF)—there was one stark difference: most of the (male) professors smoked cigarettes, often and robustly. One of my favorite hosts—a barrel-chested combat veteran—offered an explanation for this apparent paradox: “We know that smoking is bad for us, but when the future of your country is uncertain, it’s hard to abandon current pleasures for potential future benefits”.

## Progress and challenges

In the almost three decades since that encounter, it is clear that Israeli attitudes and practices about smoking have come a long way, paralleling the impressive declines in smoking in the rest of the developed world [1, 2]. Probably no other trend has contributed more to the health of the Israeli population [3]. Yet, as the paper by Feldman illustrates, there is still more to do [4]. For one, although the smoking rates found among medical (6.9 %), dental (11.6 %) and pharmacy (8.7 %) students are substantially below the 19.8 % of the general population, they are higher than health professional smoking prevalence rates in the United States, where, for example, physician smoking rates hover between 1–2 % [5–7]. Another difference is the ambivalence about the acceptability of smoke-free areas. Like the Ein Kerem campus of Hebrew University, UCSF is also a stand-alone health science campus. But it exists in a state—California—where smoking has become progressively de-normalized to the extent that social norms now suffice to enforce non-smoking bans. When UCSF became smoke-free, the only controversy was that smokers migrated off campus and left cigarette butts in residential neighborhoods. The ban was almost universally accepted, and official enforcement was not necessary.

Feldman’s survey of school of pharmacy staff and health science students revealed incomplete enforcement of the Ein Kerem campus non-smoking ban. In his discussion, he labels the ban as “an intrusive lifestyle intervention,” and speculates whether the time and politics are sufficient to justify such coercion. The assertion that freedom to smoke was a personal right undergirded campaigns of the tobacco industry in opposition to clean indoor air laws in the United States several decades ago. Ultimately, that argument was defeated by the desire of non-smokers to avoid exposure to second-hand smoke. Surprisingly, many surveys also found that a majority of smokers (though fewer than non-smokers) also supported such ordinances. Feldman articulates many reasons why such a ban is needed: second hand smoke exposure avoidance; appropriate role modeling for other health professionals as well as patients and their families; and its efficacy as a tobacco control policy. Another benefit is avoidance of extensive litter from used cigarette butts. If the experience of the United States and European countries is any guide, acceptance of smoke-free bans will not be as difficult as Feldman fears, and the enforcement will be pretty much self-governing.

## Conclusions

I just wish that my Israeli host had lived long enough to witness these changes. I hope he would take pride in the ongoing de-normalization of smoking in Israel.

## Abbreviations

UCSF, University of California San Francisco
